# Cardiovascular Dysfunction Following Burn Injury: What We Have Learned from Rat and Mouse Models

**DOI:** 10.3390/ijms17010053

**Published:** 2016-01-02

**Authors:** Ashley N. Guillory, Robert P. Clayton, David N. Herndon, Celeste C. Finnerty

**Affiliations:** 1 Department of Surgery, University of Texas Medical Branch, 301 University Blvd., Galveston, TX 77555, USA; asguillo@utmb.edu (A.N.G.); dherndon@utmb.edu (D.N.H.); 2 Shriners Hospitals for Children^®^—Galveston, 815 Market St. Galveston, TX 77550, USA; 3 Institute for Translational Sciences, University of Texas Medical Branch, 301 University Blvd., Galveston, TX 77555, USA; rpclayto@utmb.edu

**Keywords:** burns, thermal injury, cardiac dysfunction, animal models

## Abstract

Severe burn profoundly affects organs both proximal and distal to the actual burn site. Cardiovascular dysfunction is a well-documented phenomenon that increases morbidity and mortality following a massive thermal trauma. Beginning immediately post-burn, during the ebb phase, cardiac function is severely depressed. By 48 h post-injury, cardiac function rebounds and the post-burn myocardium becomes tachycardic and hyperinflammatory. While current clinical trials are investigating a variety of drugs targeted at reducing aspects of the post-burn hypermetabolic response such as heart rate and cardiac work, there is still a paucity of knowledge regarding the underlying mechanisms that induce cardiac dysfunction in the severely burned. There are many animal models of burn injury, from rodents, to sheep or swine, but the majority of burn related cardiovascular investigations have occurred in rat and mouse models. This literature review consolidates the data supporting the prevalent role that β-adrenergic receptors play in mediating post-burn cardiac dysfunction and the idea that pharmacological modulation of this receptor family is a viable therapeutic target for resolving burn-induced cardiac deficits.

## 1. Introduction

Approximately 486,000 burn injuries required medical attention in the United States in 2014, with 40,000 hospitalizations and nearly 3000 burn-related deaths [1]. Burn trauma induces a hypermetabolic, hyperinflammatory state that is characterized by muscle protein catabolism, immune dysfunction, and organ failure [2]. In pediatric patients, this state along with elevations in catecholamine levels lasts up to three years after injury and is associated with changes in normal cardiac function [3,4]. For nearly five decades, burn-induced cardiac dysfunction including increased cardiac work, tachycardia, systolic dysfunction, and elevated energy expenditure has been documented [5]. Many of these perturbations can be attributed to cardiac β-adrenergic receptors (β-ARs) being activated by circulating catecholamines, which regulate cardiac function as well as stimulate signaling pathways involved with apoptosis, inflammation, proliferation, and glucose homeostasis [6]. Increased catecholamine levels and the resultant hyperactivation of β-ARs are associated with cardiac hypertrophy and dysfunction in other patient populations [7,8].

Severe burn injury has profound effects on the entire body, often leading to multi-organ dysfunction. The human cardiac response to burn injury is characterized by two distinct events dubbed the ebb and flow phases. Patients suffering from severe burn injury experience depression of cardiac contractility and output for the first 24 to 48 h following the injury, which is referred to as the ebb phase. However, by three days post-burn, patients enter the flow phase, in which energy expenditure, heart rate, and cardiac work are elevated for more than a year after injury [9,10]. In pediatric burn patients, cardiac dysfunction has been associated with poorer outcomes. Branski and colleagues confirmed the timing of the ebb and flow phases and increased cardiac output with elevated heart rate in pediatric burn patients via transpulmonary thermodilution monitoring device (PiCCO) [11]. Half of pediatric burn patients developed systolic dysfunction, which correlated with longer hospital stays and more surgical interventions [12]. Furthermore, in infants sustaining large burn injuries, right heart failure was the most common cause of death [13]. At our pediatric burn center, 40% of massively burned patients who died from shock also had cardiovascular failure [14]. In patients who survive the burn injury, elevated catecholamine levels are associated with dramatically increased heart rate and oxygen demand that lasts for more than one year post-burn [15,16].

Animal studies have been used to expand our understanding of burn injury at the molecular level and identify mediators of post-injury cardiac dysfunction. Multiple studies in various rodent and isolated cardiomyocyte models have confirmed that depressed cardiac contractility begins almost immediately after burn injury, continues for approximately 36 h, and resolves by 72 h post-injury (*i.e.*, the ebb phase) [17,18,19,20]. However, there is a paucity of studies investigating the signaling changes associated with the flow phase.

Researchers have used various animal models to explore the complexity of burn injury pathophysiology (reviewed in [21]). Due to the complex nature of the body’s response to such a severe injury, even large animal models such as sheep or pig do not completely replicate human pathophysiology. Despite this caveat, the smaller animal models, particularly the rat and mouse models, have proven useful for investigating the mechanisms underlying the pathological manifestations of burn injury. There are two primary methods utilized in order to generate a burn injury in rodents: Contact with a heated metal probe applied to shaved skin and scald, where the animal is placed in a protective mold with the shaved area to be burned exposed to hot water [22,23]. In both models, animals are anesthetized prior to injury with either a ketamine/xylazine injection or inhaled isoflurane. The percentage of total body surface area (TBSA) burned can be adjusted by altering the number of contacts with the probe or the size of the exposed area in the mold. In the mouse model, the TBSA burned is limited to less than 40%, reducing the utility of this model as the hypermetabolic response is only invoked with larger burns (>50% TBSA burned) [24]. However, the rat model can sustain a 60% TBSA burn inducing a clinically relevant injury and subsequent hypermetabolic response similar to those seen in severely burned patients [23]. While the principles of these models have been standardized, a great deal of variability exists in the experimental design from study to study including the temperature of the water or probe, the length of burn administration, and the amount of resuscitation fluid, which can all alter post-burn outcomes. Despite these differences, the utilization of these models has greatly advanced our understanding of burn induced signaling changes in the heart and other organs.

## 2. Molecular Mechanisms Underlying Burn-Induced Cardiac Dysfunction

### 2.1. β-Adrenergic Receptors

We propose that many of the characteristic cardiac perturbations following burn-induced, persistent elevations in catecholamine release can be attributed to the β-ARs. Although their primary function is to modulate cardiac chronotropy, lusitropy, and inotropy through the movement of calcium, these receptors also regulate apoptosis, inflammation, proliferation, and glucose homeostasis. The β-ARs also participate in cross-talk with a variety of signaling receptors including the androgen receptor and the peroxisome proliferator-activated receptor α [25,26,27]. Through these and other signaling mechanisms, prolonged activation of the β-ARs results in increased morbidity and mortality [2]. Because of the widespread influence of β-ARs on cardiac function and morphology, pharmacological modulation of β-AR signaling may attenuate the majority of pathological signaling that occurs following burn injury.

**Figure 1:**
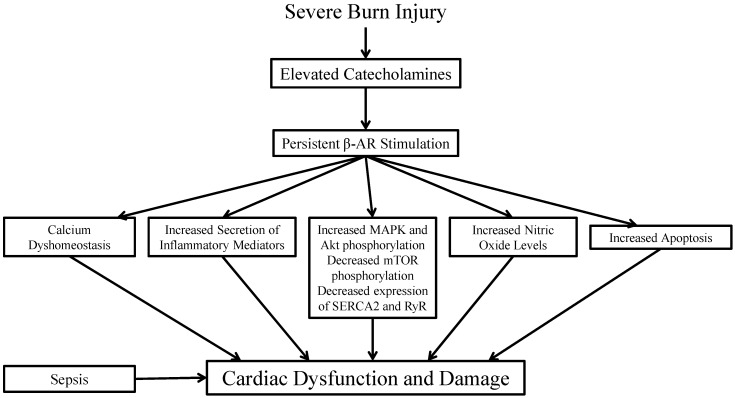
Schematic of the signaling changes that are postulated to occur downstream of β-adrenergic receptor (β-AR) stimulation following burn injury. MAPK, mitogen-activated protein kinase; Akt, protein kinase B; mTOR, mammalian target of rapamycin; SERCA2, sarcoplasmic reticulum calcium ATPase 2; RyR, ryanodine receptor.

Prolonged catecholamine release can lead to uncoupling of β-ARs from G proteins, decreased 3′-5′-cyclic adenosine monophosphate (cAMP) production, and altered phosphorylation and expression of proteins involved in various downstream pathways (Figure 1). For example, p38, jun N-terminal kinase (JNK), and extracellular signal-regulated kinase 1/2 (ERK1/2) mitogen-activated protein kinase (MAPK) phosphorylation was significantly increased in the first 4 h after injury [28,29,30]. Similarly, Akt (protein kinase B) phosphorylation was also increased shortly after burn injury (peaked at 3 h) [31]. Protein expression of both the sarcoplasmic reticulum calcium ATPase 2 (SERCA2) and ryanodine receptor was significantly reduced after burn injury [32]. The mammalian target of rapamycin (mTOR) phosphorylation was also significantly reduced after burn injury [33]. Many of these proteins are involved in modulating the influx of calcium through depolarized calcium channels, calcium-induced calcium release from the sarcoplasmic reticulum, and subsequent removal of intracellular calcium by various measures. This movement of calcium in and out of the intracellular space can be graphically represented by what is referred to as a calcium transient [6]. Using cardiomyocytes isolated from burned rats, Koshy *et al.*, observed that calcium transients were unresponsive to additional β-AR stimulation, indicating desensitization of the receptors [34]. Earlier studies determined that β-AR affinity in rat ventricular muscle was significantly decreased 7 days and 14 days after burn injury. At these time points, cAMP content was also decreased. Similar to what was seen in the calcium transients, isoproterenol-stimulated cAMP production was decreased 24 h and 14 days post-injury [35]. Furthermore, Zhang *et al.*, reported that burn injury depressed expression of stimulatory G protein (Gs) mRNA as early as 3 h post-injury [36]. Thus, burn-induced depression of cardiac function may be attributed to the inability of β-ARs to respond to additional stimuli. There remains controversy regarding whether the inability to respond is due to decreased receptor density or to uncoupling of the receptors from their G protein partners. However, to date, there are no published studies investigating whether uncoupling occurs, and there are conflicting results regarding whether β-AR density is altered post-burn. Surprisingly, pretreatment with clonidine, an α adrenergic receptor (α-AR) agonist, blocked burn-induced downregulation of β-AR density, adenylyl cyclase activity, and cAMP content. However, whether clonidine restored cardiac function was not assessed, nor was the mechanism involved in upregulation of β-AR density [37].

### 2.2. Cytokines and Pro-Inflammatory Mediators

The majority of research investigating post-burn cardiac signaling changes in animal models has focused on the inflammatory response. This response can also be regulated by β-AR-dependent signaling, although other receptor populations also contribute. However, many of these inflammatory pathways cross-talk with β-ARs, providing additional evidence that β-ARs are key mediators of burn-induced cardiac dysfunction. Interleukin (IL)-1β and tumor necrosis factor α (TNFα) expression and secretion are regulated by nuclear factor κ B (NFκB) and p38 MAPK which can be activated following β-AR stimulation [38,39,40]. These factors have been shown to be upregulated in the heart following burn injury (Figure 1) [41]. TNFα secretion was increased more than 10-fold in cardiomyocytes from burned animals [17]. Inhibition of NFκB translocation into the nucleus by IκB prevented TNFα secretion as well as normalized cardiac function after burn injury. In addition to inducing inflammation, TNFα perfusion into normal hearts was sufficient to severely depress myocardial function [40]. Depressed cardiac function was also observed following IL-1β treatment despite the absence of TNFα [42]. However, maximal cardiac dysfunction occurred with co-administration of IL-1β, TNFα, and IL-6 [43].

Toll-like receptor 4 (TLR4) also contributes to the maladaptive cardiac inflammatory response to severe burn injury. The aforementioned burn-induced inflammatory cascade was significantly blunted in severely burned mice genetically engineered to produce an inactive form of TLR4 [41]. The same authors also investigated the role of cluster of differentiation 14 (CD14), an endotoxin receptor that forms a complex with TLR4. CD14 knockout significantly reduced the production of IL-6, TNFα, and IL-1β after burn injury. Furthermore, left ventricular function was restored, as seen through comparison to burned wildtype mice. Similar results were noted using pharmacological inhibition of CD14 [44]. Lipopolysaccharide (LPS) and endotoxin release also contribute to burn induced contractile dysfunction and inflammation. In cardiomyocytes isolated from burned rats, LPS administration decreased sarcomere shortening in a dose-dependent manner, indicating impairment of cardiomyocyte contraction [45]. The effect of LPS on sarcomere shortening was independent of burn injury, as burn injury with LPS treatment had an additive effect [45]. Recombinant bactericidal permeability-increasing protein (BPI) attenuated the burn pathophysiology [46]. These data indicate that the relationship between inflammation and cardiac dysfunction may yield new targets for future clinical investigations.

### 2.3. Nitric Oxide

In animal models of both ischemic-reperfusion injury and sepsis, nitric oxide (NO) is a negative inotrope. Burn trauma also augments NO levels primarily through upregulation of inducible nitric oxide synthase (iNOS; Figure 1). Increased NO levels lead to oxidative stress causing DNA breakage and activation of NFκB [47]. Inflammatory cytokines such as TNFα can also promote iNOS activity. Cytokine activation of iNOS leads to a persistent elevation of NO levels, which can contribute to cytotoxicity and ventricular dysfunction [48].

Cardiac-specific knockout of iNOS dampened the burn-induced cardiovascular deficits observed in wildtype mice. Similar results were obtained using pharmacological inhibition of iNOS [49]. Mitochondrial NOS levels were also significantly increased after severe burn injury [50]. Elevated NO levels mediate the formation of compounds that alter mitochondrial respiration and compete with calcium for binding sites on contractile proteins. The formation of these compounds can severely depress cardiac function [51,52,53]. Conversely, NO also performs a vital role in cell survival by scavenging free radicals and inhibiting bacterial invasion when levels are relatively low [54]. Thus, the circulating concentration of NO dictates the observed effects, and overcompensation in either direction can significantly contribute to cardiac dysfunction.

β_3_-AR signaling is one of the regulators of cardiac NO levels. Activation of β_3_-ARs may lead to NFκB translocation and promotion of hypertrophic, apoptotic, and inflammatory signaling [55]. NFκB translocation can also occur in response to β_1_- and β_2_-AR stimulation. Furthermore, NFκB suppression dampens β-AR-dependent hypertrophy [56]. These data further implicate β-ARs as a key component of impaired cardiac function following burn injury.

### 2.4. Calcium

Calcium homeostasis is vital for maintaining cardiomyocyte function and viability. The entry, extrusion, and storage of calcium dictate the rate and force of contractility as well as the rate of relaxation. There is a plethora of evidence relating changes in calcium handling to cardiac hypertrophy and failure, and numerous studies indicate that altered calcium handling occurs following burn injury and correlates with negative inotropy (Figure 1) [32,34,57,58]. Indeed, treatment of isolated cardiomyocytes with burn serum in the 40% TBSA burned rat model induced hyperactivity of ryanodine receptors, the calcium release channels that reside on the sarcoplasmic reticulum. Hyperactive ryanodine receptors initially increase calcium release, augmenting contractility. However, prolonged leakage of calcium from the sarcoplasmic reticulum depletes calcium stores, resulting in depressed contractility [59,60]. Severe burn injury also decreases l-type calcium channel protein expression, thus reducing intracellular calcium influx [61]. More recent studies have found that burn injury decreases calcium current and contractility despite maintenance of l-type calcium channel mRNA levels [62].

Calcium is highly cytotoxic and accumulation of intracellular calcium can lead to derangement of signaling pathways, release of cytokines, and ventricular dysfunction. Post-burn calcium accumulation is blocked by inhibition of protein kinase C (PKC). Moreover, burn-induced perturbations in ventricular function are reduced by inhibiting PKC [63]. Cytosolic calcium accumulation also occurs in a 2-hit model of burn with sepsis [64]. Increased calcium uptake into mitochondria can contribute to myocardial inflammation and injury. Maass *et al.*, isolated cardiomyocytes from severely burned rats and showed that calcium accumulated in both the cytosol and the mitochondria. This mitochondrial calcium overload also played a critical role in the release of TNFα, IL-1β, and IL-6 [65]. Mitochondrial calcium levels significantly increase 1 h post-burn and remain elevated 24 h post-burn [50]. Evidence of calcium overload prior to ventricular contractile dysfunction suggests that calcium dyshomeostasis contributes to the induction of myocardial injury and dysfunction [32]. After burn injury occurs, Ca^2+^/Mg^2+^-ATPase and Na^+^/K^+^-ATPase activity is reduced, potentially increasing intracellular calcium accumulation [36]. Calcium antagonists blunt burn-induced calcium accumulation, TNFα secretion, and impairment of left ventricular function [58].

Concentrations of cytosolic free calcium have been shown to increase significantly in cardiomyocytes isolated 24 h after burn injury. However, additional stimulation with isoproterenol, a β-AR agonist, does not alter the time course of the calcium transient. These data suggest that desensitization of β-ARs may contribute to calcium dyshomeostasis [34]. In other models of disease, treatment with β-AR antagonists resolves calcium signaling alterations caused by elevated catecholamine levels [66]. However, the effect of β-blockade on calcium handling can differ depending on the antagonist used [67].

### 2.5. Apoptosis

Cell death by apoptosis is a highly organized and regulated process that is tailored to organismal development and growth; unregulated apoptosis can impair organ function. Cardiac apoptosis has been detected as early as 6 h post injury, and markers of apoptosis such as DNA fragmentation and caspase-3 activity have been noted 3 to 12 h post-burn (Figure 1). Increased apoptosis correlates with defects in cardiac contraction and relaxation [68,69]. Increased intracellular calcium such as is seen following burn injury can trigger apoptosis in a multitude of tissues [58,70]. Mitochondria also contribute to burn-induced apoptosis by releasing apoptosis-inducing factor and cytochrome c and into the cytoplasm [71].

In a 40% TBSA burned rat model, upregulation of both apoptosis and caspase-3 activity (a common marker of apoptosis) occurs. Pharmacological inhibition of extracellular regulated kinase and phosphoinositide-3 kinase further exacerbates cardiac apoptosis [30]. Similarly, others have shown that burn injury promotes apoptotic signaling by inhibiting Akt (protein kinase B) while activating p38 MAPK [72,73]. Attenuation of apoptosis by inhibition of p38 MAPK reduces expression of TNFα and iNOS and decreases oxidative stress following burn injury [74,75]. Inhibition of endotoxin activity using a recombinant N-terminal fragment of BPI can also prevent the upregulation of caspase-3 activity caused by burn plasma administration to murine cardiomyocytes [69].

As mentioned previously, β-AR signaling can mediate apoptosis. β_1_-AR primarily promotes apoptosis while activation of β_2_-AR signaling has an anti-apoptotic effect [76]. β_1_-AR-induced apoptosis can occur independently of protein kinase A (PKA) activity [77]. MAPK activation and TNFα secretion can be regulated by β-ARs [28,38,40]. Thus, β-AR signaling may contribute to mechanisms underlying burn-induced apoptosis.

### 2.6. Sepsis

In a single institution study at the Shriners Hospital for Children^®^—Galveston, more than 30% of burn patient deaths were attributed to sepsis [14]. Investigation of the molecular mechanisms underlying sepsis can be difficult as most animal models of large burn injury alone do not develop sepsis. Using various 2-hit models to mimic burn complicated by sepsis, Horton *et al.*, showed that sepsis exacerbates both the inflammatory response and ventricular dysfunction in rats with 40% TBSA burns [17,78]. As confirmed in a mouse 2-hit model, cardiac function is further dampened in the presence of sepsis [79]. The type of infectious organism alters the host response; a gram-negative challenge results in a higher mortality rate than a gram-positive challenge [80].

Decreased levels of the sarcoplasmic reticulum calcium ATPase in a burn-complicated-by-sepsis model indicate that calcium dyshomeostasis may contribute to the development of sepsis [64]. The addition of cecal ligation and puncture to a 30% TBSA scald burn rat model resulted in an initial augmentation of cardiac function that progressed into a hypodynamic state along with an increased mortality rate [81]. Time elapsed between the injury and the development of sepsis may also contribute to the severity of the cardiac response. In a study comparing aspiration pneumonia-induced sepsis induced 48 or 72 h post-burn, earlier induction of sepsis augmented cardiac contractile dysfunction. These changes were not seen when sepsis was induced 72 h following the burn injury; these animals had similar cardiac function relative to the burn only group [18]. While there is not a clear connection between β-AR signaling and the induction of sepsis, it can be appreciated that β-AR-dependent calcium dyshomeostasis and inflammation can contribute to the susceptibility to and progression of sepsis.

### 2.7. Pharmacological Modulation

A variety of pharmaceutical agents are administered to attenuate the burn-induced hypermetabolic response and subsequent hypercatabolism (Table 1; reviewed in [82,83]). The majority of investigational pharmacological approaches have utilized anabolic agents (e.g., insulin, growth hormone, oxandrolone, insulin-like growth factor) to increase lean body mass retention. Administration of insulin (with various dosing regimens), fenofibrate, metformin, or glucagon-like peptide has resulted in attenuation of hyperglycemia and insulin resistance, additional characteristics of the hypermetabolic response. While many of these pharmacological modulations target components of the β-AR signaling pathway, few investigators have explored the use of β-blockade to mitigate burn-induced cardiac dysfunction. There is strong evidence that propranolol, a nonspecific β-blocker, improves cardiac outcomes in severely burned pediatric patients. Propranolol administration was initially proposed because it had previously been used safely in other pediatric patient populations to decrease heart rate. Since propranolol blocks both of the major cardiac β-AR subtypes, in addition to reducing heart rate and cardiac work (primarily mediated through β_1_-AR), propranolol administration dampens other hypermetabolic, hypercatabolic responses to burn injury such as increased resting energy expenditure, lipolysis (β_2_-AR dependent signaling), and protein breakdown [2,84,85,86,87,88]. This decrease in peripheral lipolysis was not observed when metoprolol, a β_1_-AR selective blocker, was used [89].

**Table 1:** Recent pharmacotherapeutic agents used in the treatment of burn injury.

Drug	Clinical Reference(s)
Propranolol	[2,84,85,86,87,88,90,91,92,93,94]
Oxandrolone	[95,96,97,98,99,100,101,102,103,104,105]
Fenofibrate	[106,107,108]
Growth Hormone	[16,92,109,110,111,112,113,114,115,116,117,118,119,120,121,122,123]
Insulin	[124,125,126,127,128,129,130]
Insulin-Like Growth Factor 1	[131,132,133,134,135,136,137]
Ketoconazole	[138]
Metformin	[139,140,141]

Many of the aforementioned therapeutics have cardiovascular activity in addition to their primary mechanism of action. For example, insulin administration has been shown to inhibit caspase-3, bcl2, and p38 MAPK in burn serum-challenged cardiomyocytes, dampening burn-induced apoptosis. In the same study, insulin administration attenuated inflammatory cytokine release [72]. Although insulin does not directly bind to β-ARs, studies in cardiomyocytes reveal that insulin recruits GRK2 to β_2_-ARs to modulate cAMP/PKA-dependent signaling [142]. Thus, the anti-apoptotic effects of insulin may be partially attributed to insulin mediated inhibition of β_2_-ARs. As previously mentioned, activation of p38 MPAK and the caspases alongside generation of reactive oxygen species can occur downstream of β-ARs, and all have been implicated in pro-inflammatory signaling cascades. Accordingly, p38 MAPK inhibition rescues the burn-induced inflammatory response [74,75]. Similarly, improvement of cardiac function can be obtained by pretreatment with caspase inhibitors, which reduces release of inflammatory cytokines [143]. Antioxidant vitamin therapy also decreases the nuclear translocation of NFκB and TNFα, IL-1β, and IL-6 secretion from cardiomyocytes [144]. Further studies are needed to identify novel therapeutic targets and to determine the best pharmaceutical agent for the attenuation of burn-induced cardiac dysfunction.

Despite the overwhelming clinical data supporting the use of propranolol administration after burn injury, the few preclinical studies that have been conducted have yielded conflicting results. Propranolol pretreatment (3 mg/kg/day for 14 days prior to burn) in a guinea pig model, resulted in a greater need for fluid resuscitation compared to vehicle-treated animals perhaps due to the pretreatment predisposing the animals to depressed cardiac function [145]. Propranolol (0.75 mg/kg) given 30 min after the burn injury (0.5 h) also significantly worsened cardiac function and contributed to hypoperfusion-induced organ damage [146]. The timing of the administration of propranolol was not ideal as cardiac depression occurs in response to both the anesthesia and the injury. By giving propranolol so soon after the injury, it is possible that the animals had not recovered sufficiently to experience the hypermetabolic response that propranolol should treat. Thus, these differences between the clinical and preclinical studies may be primarily attributed to the study design. Ongoing investigations by our group and others will further elucidate the molecular mechanisms of burn induced cardiac dysfunction to better target therapeutic interventions.

## 3. Limitations and Confounding Factors

Cardiac dysfunction and the post-burn hypermetabolic and hyperinflammatory states persist for more than a year post injury in pediatric patients, well after visible healing of the injury. However, there are very few studies with experimental designs investigating cardiac dysfunction more than 24 h post injury. Of note, many of the animal models from earlier studies did not include resuscitation (as is used in the patients) whereas in later studies included resuscitation with lactated Ringer’s solution. Resuscitation protocols also varied from one bolus of resuscitation fluids administered immediately after burn to continued administration of fluids for up to 12 h post-burn. This change in resuscitation practice may explain the reduced severity and persistence of cardiac dysfunction in later studies. Use of multiple rat and mouse strains can also confound interpretation of the role and extent of post-burn cardiac dysfunction. The size of the burn injury varied from 20% to 50%; as larger burns are associated with cardiac and metabolic complications while smaller burns are not, these variations hinder direct comparison of data and may contribute to the lack of agreement regarding the timing of apoptosis, cytokine release, and cardiac dysfunction. Similarly, the fact that sepsis models rely on different times of induction and a wide range of microorganisms for sepsis induction hinders definitive interpretation of the effect of sepsis on burn-induced perturbations in cardiac signaling.

While a substantial amount of work has been summarized in this review, it is clear that there are still some significant gaps in knowledge and new avenues of research to investigate. Over the last decade there have been great advances in G-protein coupled receptor signaling that extend far beyond cAMP/PKA dependent signaling, however, most of the burn related research has focused on this canonical β-AR signaling pathway. Epac mediated cAMP signaling and β-arrestin dependent signaling are just two possible noncanonical signaling pathways contributing to cardiac dysfunction induced by a burn injury. Epac signaling can affect intracellular calcium handling and interleukin 6 release, both of which has been shown to be altered after severe burn injury [147,148]. The cardioprotective effects of β-arrestin dependent signaling in heart failure have been demonstrated and it would be worthwhile to determine whether this signaling cascade is activated post-burn as well as determine the effect of therapies such as propranolol [149,150].

Biased signaling is another recently elucidated concept that should be considered both during experimental design as well as during interpretation of results [151,152]. This concept operates on the premise that different ligands can turn on or shut off specific effectors despite binding to the same receptor. As mentioned previously, propranolol is the primary β-blocker that has been investigated in the context of burn injury. While at least one small study was conducted examining metoprolol treatment after burn injury, there are no published results utilizing any of the newer β-blockers in either pre-clinical or clinical studies. Carvedilol holds particular interest as it has α-AR blocking activity in addition to its nonspecific blockade of β-ARs. Preclinical studies indicated that pretreatment with α-AR agonist clonidine was beneficial in dampening the effect of burn injury on β-ARs [37]. Thus, it is unclear whether carvedilol would improve or depress post-burn cardiac signaling and function. Furthermore, the role of α-ARs in burn injury has not been well studied. These receptors also respond to stimulation by circulating catecholamines and are involved in modulating cardiac function and may be responsible for some of the observations attributed to β-ARs [153]. As we increase our knowledge of burn induced cardiac dysfunction and its underlying mechanisms, biased signaling can be utilized to determine the best pharmacological treatment to improve patient outcomes and minimize adverse events.

## 4. Conclusions

Cardiovascular dysfunction following burn injury is a major problem in humans and can be replicated and studied in animals. A rapid and robust inflammatory response precedes the onset of defects in left ventricular pressure and sarcomere shortening. Calcium dyshomeostasis and dysregulation of NO levels also contribute to depression of cardiac contractility. Sepsis or infection further exacerbates cardiac depression. Many of these signaling changes occur downstream of β-ARs and may be the result of overstimulation of these receptors by elevated circulating catecholamine levels. Figure 1 summarizes the studies examined in this review and depicts how β-ARs may be the impetus for these signaling changes and the induction of cardiac dysfunction and damage.
